# Guanidinoacetic Acid and Cysteamine Hydrochloride Improve Beef Cattle Growth and Antioxidant Capacity via Fecal Microbiome and Serum Metabolome

**DOI:** 10.3390/antiox15091178

**Published:** 2026-09-16

**Authors:** Baoshan Feng, Hongwei Zhang, Jianjun Guo, Meishu Tan, Sen Mao, Bin Yu, Yiran Xu, Yanan Wang, Man Feng, Lianjie Song, Yinghao Zhou, Hongjian Xu

**Affiliations:** 1 College of Animal Science and Technology, Hebei Agricultural University, Baoding 071001, China; ashan0711@163.com; 2 Chengde Academy of Agriculture and Forestry Sciences, Chengde 067000, China; nkyzhanghongwei@126.com (H.Z.); gjj730209@126.com (J.G.); tanmw0415@163.com (M.T.); maosen2026@163.com (S.M.); cdsxmyjs@163.com (B.Y.); xyr18730402787@163.com (Y.X.); tzhy8566@163.com (Y.W.); fengman59@163.com (M.F.); songlianjie1993@163.com (L.S.)

**Keywords:** guanidinoacetic acid, cysteamine hydrochloride, growth performance, antioxidant capacity, fecal microbiome, serum metabolomics

## Abstract

Balancing muscle growth and antioxidant/anti-inflammatory homeostasis is a core requirement for modern beef production. This study investigated whether cysteamine hydrochloride (CSH) co-supplementation with guanidinoacetic acid (GAA) enhances growth and antioxidant status through fecal microbiome and serum metabolome modulation in Simmental beef cattle. A 60-day trial randomly assigned 45 10-month-old cattle to control (CON), GAA-supplemented, and GAA + CSH-supplemented groups (*n* = 15 per group). Growth performance was recorded throughout the trial. Fecal samples were collected for 16S rRNA sequencing, and serum samples for non-targeted metabolomics. Both GAA and GAA + CSH (GCSH) improved growth performance versus CON (*p* < 0.05), with no differences between supplemented groups. However, GCSH uniquely elevated antioxidant enzymes and reduced malondialdehyde (*p* < 0.05). GCSH treatment was associated with higher abundance of *Adlercreutzia*, *Anaerofustis*, and *Monoglobus*, alongside elevated indole-3-propionic acid and salicylsulfuric acid. In contrast, GAA reduced phenylacetyl-L-glutamine and indole-3-acetic acid. iCAMP analysis revealed that GCSH relieved homogenizing selection while enhancing heterogeneous selection, promoting beneficial taxa colonization. GAA primarily enhances nitrogen utilization, while CSH enriches beneficial microbiota and antioxidant metabolites. These findings suggest that GAA and CSH co-supplementation improves growth and antioxidant homeostasis through complementary mechanisms.

## 1. Introduction

In the modern beef cattle industry, optimizing muscle growth and lean meat yield remains a central production goal, driven by the ever-increasing global demand for high-quality beef. Skeletal muscle accretion depends on a delicate balance between protein synthesis and degradation, a process tightly regulated by nutritional, hormonal, and cellular energy status [1]. However, under intensive production conditions, cattle are frequently exposed to various stressors that disrupt this balance, among which oxidative stress represents a key limiting factor. Excessive accumulation of reactive oxygen species (ROS) triggers lipid peroxidation, protein oxidation, and DNA damage, ultimately impairing muscle protein deposition, immune function, and overall feed conversion efficiency [2]. Therefore, enhancing the endogenous antioxidant defense system through nutritional strategies holds both scientific significance and practical value for sustaining muscle growth and improving beef cattle performance.

Guanidinoacetic acid (GAA), the sole precursor of creatine biosynthesis, enhances intramuscular creatine stores, optimizes energy metabolism, and improves growth performance in ruminants [3,4]. By supplying the guanidino group for creatine synthesis, GAA spares arginine from this pathway, redirecting it toward protein synthesis, nitric oxide production, and immune function, thereby supporting nitrogen retention and muscle accretion. However, GAA acts primarily through metabolic pathways and does not directly engage the antioxidant or immune systems. Cysteamine hydrochloride (CSH), a sulfhydryl-containing compound, offers a functionally complementary profile. CSH enhances endogenous antioxidant capacity by donating sulfhydryl groups, promoting glutathione synthesis, and upregulating antioxidant enzyme activities. In parallel, CSH modulates immune signaling by suppressing pro-inflammatory cytokine release and maintaining intestinal barrier integrity [5,6,7]. These immunomodulatory and redox-balancing properties position CSH as a natural partner to GAA: while GAA builds the engine of muscle energy metabolism, CSH reinforces the cellular defense and immune surveillance systems that sustain long-term metabolic health. Given that the fecal microbiome critically influences host redox homeostasis and that dietary additives can be metabolized by gut microbes to produce signaling molecules that modulate systemic inflammation, whether combined GAA and CSH supplementation enhance antioxidant defense through fecal microbiome remodeling and its metabolic output remains unexplored.

It was hypothesized that GAA supplementation would improve growth performance in beef cattle, that CSH co-supplementation would confer additional antioxidant and anti-inflammatory benefits, and that this would be associated with distinct remodeling of the fecal microbiome and serum metabolome. To test this hypothesis, a 60-day feeding trial was conducted with Simmental beef cattle randomly assigned to three groups: control, GAA-supplemented, and GAA + CSH-supplemented. Growth performance, nutrient digestibility, serum antioxidant parameters, fecal 16S rRNA microbiome, and serum metabolomics were integrated to unravel the mechanisms of GAA and GAA + CSH supplementation. This study aims to provide a theoretical basis and new insights for precision nutritional regulation in beef cattle.

## 2. Materials and Methods

### 2.1. Animals, Diets, and Experimental Design

All animal procedures were approved by the Institutional Animal Care and Use Committee of Hebei Agricultural University (Permit Number: 2025032) and conducted at the Longhua County Fuqiang Planting and Breeding Professional Cooperative (Chengde City, Hebei Province, China).

A total of 45 10-month-old Simmental beef cattle (body weight = 285.00 ± 23.64 kg) were used in a 60-day feeding trial. All cattle were sourced from the same farm and were of comparable age. On the day of arrival, cattle were weighed and immediately randomly assigned to one of three dietary treatments (*n* = 15 per treatment) based on their body weight to ensure balanced group allocation: (1) control group—fed a basal total mixed ration (TMR); (2) GAA group—fed the basal TMR supplemented with guanidinoacetic acid at 0.55 g/kg dry matter (DM); (3) GAA + CSH (GCSH) group—fed the basal TMR supplemented with the same dose of GAA plus 0.05 g/kg cysteamine hydrochloride (DM basis). The sample size (15 animals per group) was determined based on the typical group sizes used in beef cattle nutrition studies and was considered sufficient to detect biologically meaningful differences in growth performance. All feed additives were provided by Ringpu Biotechnology Co., Ltd. (Tianjin, China). The basal diet was formulated to meet or exceed the nutrient requirements for growing and finishing beef cattle according to the National Research Council [8]. The ingredients and chemical composition of the basal diet are shown in Table 1. Cattle were individually housed in barns and had ad libitum access to feed and fresh water. TMR was offered twice daily at 7:00 AM and 7:00 PM, and feed refusals were collected and weighed daily before the morning feeding to determine dry matter intake (DMI).

### 2.2. Growth Performance Measurement

Body weight was recorded on day 0, day 30, and day 60. Average daily gain (ADG) was calculated for each period (days 0–30, days 31–60, and days 0–60) as the difference between final and initial body weight divided by the number of days in each period. Daily feed intake was recorded individually, and DMI was calculated based on daily feed intake and the dry matter content of the offered feed. Feed efficiency was expressed as the feed conversion ratio (FCR), calculated as DMI divided by ADG.

### 2.3. Sample Collection

On day 60, before the morning feeding, blood samples were collected from each animal via jugular venipuncture. Blood samples were centrifuged at 3000× *g* for 15 min at 4 °C to obtain serum, which was aliquoted and stored at −80 °C for subsequent biochemical, antioxidant, and metabolomic analyses. Fecal samples were collected directly from the rectum on days 58–60, pooled per animal, and a portion was immediately frozen in liquid nitrogen and stored at −80 °C for 16S rRNA gene sequencing. Another portion was dried at 55 °C for 48 h for apparent digestibility determination. Samples of the basal diet were collected weekly, composited, and stored for chemical analysis.

### 2.4. Apparent Nutrient Digestibility

Apparent total-tract digestibility of nutrients was determined using acid-insoluble ash (AIA) as an internal marker. Feed and fecal samples were analyzed for dry matter (DM, method 930.15), crude protein (CP, method 996.11), acid detergent fiber (ADF), and neutral detergent fiber (NDF) according to AOAC procedures [9]. AIA content was measured following the method of Van Keulen and Young [10].

Digestibility = 100 − 100 × (Indicator% in diet × Nutrient% in feces)/(Indicator% in feces × Nutrient% in diet) [10].

### 2.5. Serum Biochemical and Antioxidant Analyses

Serum concentrations of glucose (GLU), total protein (TP), albumin (ALB), blood urea nitrogen (BUN), and total cholesterol (TC) were determined using a Mindray BS-420 automatic biochemical analyzer (Mindray Bio-Medical Electronics Co., Ltd., Shenzhen, China) with commercial colorimetric assay kits (ZhongSheng Beikong Biotechnology Co., Ltd., Beijing, China) according to the manufacturer’s protocols. Globulin (GLB) concentration was calculated as the difference between TP and ALB. Serum total antioxidant capacity (T-AOC), activities of superoxide dismutase (SOD) and catalase (CAT), and malondialdehyde (MDA) content were measured using bovine-specific enzyme-linked immunosorbent assay (ELISA) kits (Beijing Huaying Biotechnology Research Institute, Beijing, China) according to the manufacturer’s instructions. All ELISAs (T-AOC, SOD, CAT, and MDA) were performed in a single analytical batch, and all samples were analyzed in duplicate. Intra-assay coefficients of variation (CVs) were calculated based on duplicate measurements of all individual samples in this study, with observed values of T-AOC 4.2%, SOD 3.7%, CAT 3.2%, and MDA 5.1%, all within the manufacturer’s specified range (<10%). To monitor signal drift throughout a single analytical run, a pooled quality control (QC) serum sample was prepared by mixing equal aliquots from all experimental animals and assayed repeatedly at regular intervals. CVs of repeated QC measurements were calculated to evaluate the within-run stability, with observed values of T-AOC 6.3%, SOD 5.4%, CAT 4.5%, and MDA 7.2%, all below the manufacturer’s specification (<12%). Biochemical parameters (GLU, TP, ALB, BUN, TC) were determined using an automated biochemical analyzer with routine quality control procedures. Under this system, the observed CVs for all routine biochemical parameters were <3.0%.

### 2.6. Fecal 16S rRNA Gene Sequencing and Analysis

Microbial genomic DNA was extracted from 0.5 g of fecal samples using the E.Z.N.A.^®^ Soil DNA Kit (Omega Bio-tek, Norcross, GA, USA) according to the manufacturer’s instructions. The DNA integrity, concentration, and purity were assessed by 1% agarose gel electrophoresis and a NanoDrop 2000 spectrophotometer Thermo Fisher Scientific, Waltham, MA, USA).

The V3–V4 region of the 16S rRNA gene was amplified with barcoded primers 338F/806R [11] using TransStart FastPfu DNA Polymerase (TransGen Biotech, Beijing, China). The 20 μL PCR mixture contained 4 μL of 5× buffer, 2 μL of 2.5 mM dNTPs, 0.8 μL of each primer (5 μM), 0.4 μL of polymerase, and ~10 ng of template DNA. Amplification was performed at 95 °C for 3 min; 27 cycles of 95 °C for 30 s, 55 °C for 30 s, and 72 °C for 30 s; and a final extension at 72 °C for 10 min. PCR products were purified using a PCR Clean-Up Kit (Yu Hua Biotechnology Co., Ltd., Shanghai, China) after 2% agarose gel electrophoresis and quantified with a Qubit 4.0 fluorometer (Thermo Fisher Scientific, Waltham, MA, USA). Sequencing libraries were constructed with the NEXTFLEX Rapid DNA-Seq Kit (Majorbio Bio-Pharm Technology Co., Ltd., Shanghai, China) following the manufacturer’s protocol. Paired-end sequencing (2 × 300 bp) was performed on an Illumina NextSeq 2000 platform (Majorbio Bio-Pharm Technology Co., Ltd., Shanghai, China).

Raw reads were quality-filtered and merged using fastp [12] (v0.19.6) and FLASH [13] (v1.2.11) with the following criteria: (i) reads with quality scores of <20 over a 10-bp sliding window were truncated; (ii) merged reads required a minimum overlap of 10 bp and a maximum mismatch ratio of 0.2 in the overlap region; and (iii) barcodes and primers were matched with 0 and 2 mismatches allowed, respectively. The quality-controlled sequences were denoised into amplicon sequence variants (ASVs) using DADA2 [14] within QIIME2 [15]. Chloroplast and mitochondrial sequences were removed, and samples were rarefied to 20,000 reads per sample, achieving an average Good’s coverage of 99.09%. Taxonomic classification of the resulting ASVs was performed using the Naive Bayes classifier implemented in QIIME2 against the SILVA 16S rRNA gene database (release 138.2).

Downstream analyses were performed on the Majorbio Cloud Platform. Alpha diversity (Chao1 richness estimator, abundance-based coverage estimator (ACE), Shannon diversity index, and Simpson diversity index) was calculated using mothur [16] and compared among groups via the Wilcoxon rank-sum test. Beta diversity was assessed using Principal Coordinate Analysis (PCoA) based on weighted UniFrac distances for phylogenetic information, with group differences tested by permutational multivariate analysis of variance (PERMANOVA). Linear Discriminant Analysis Effect Size (LEfSe) analysis was conducted with a linear discriminant analysis (LDA) threshold of 2.0, and *p*-values were derived from Kruskal–Wallis tests without multiple-testing correction, consistent with the standard LEfSe workflow [17].

### 2.7. Serum Non-Targeted Metabolomics

Serum (100 μL) was extracted with 800 μL of ice-cold acetonitrile:methanol (1:1, *v*/*v*) containing internal standards (0.02 mg/mL L-2-chlorophenylalanine, etc.) by vortexing for 30 s and ultrasonication (5 °C, 40 kHz, 30 min). Proteins were precipitated at −20 °C for 30 min, and after centrifugation (13,000× *g*, 15 min, 4 °C), the supernatant was dried under nitrogen, redissolved in 100 μL of acetonitrile:water (1:1, *v*/*v*), ultrasonicated for 5 min, and centrifuged again (13,000× *g*, 10 min, 4 °C). The final supernatant was transferred to vials for LC-MS/MS analysis. A pooled quality control (QC) sample was prepared by mixing equal volumes of all individual samples and analyzed at regular intervals (every 5–15 runs) to monitor system stability. Analytical reproducibility was evaluated by calculating the coefficient of variation in peak intensity for all features across QC injections. The average intra-batch CV of retained metabolic features was 12.8%, with 83.4% of features showing CVs of <20%, confirming satisfactory stability of the LC-MS system. Features with QC CVs of >30% were excluded from subsequent statistical analysis, consistent with standard non-targeted metabolomics workflows.

Chromatographic separation was performed on a UHPLC-Q Exactive HF-X system (Thermo Fisher Scientific, Waltham, MA, USA) equipped with an ACQUITY HSS T3 column (100 × 2.1 mm, 1.8 μm; Waters Corporation, Milford, MA, USA) at Majorbio Bio-Pharm Technology Co., Ltd. (Shanghai, China). The mobile phases consisted of 0.1% formic acid in water:acetonitrile (2:98, *v*/*v*) (A) and 0.1% formic acid in acetonitrile (B), with a flow rate of 0.40 mL/min, a column temperature of 40 °C, and an injection volume of 5 μL. Mass spectrometry was performed in both positive and negative ESI modes under the following conditions: source temperature 400 °C; sheath gas 40 arb; aux gas 10 arb; ion-spray voltage ±3.5/−2.8 kV; collision energy 20–40–60 eV; mass range 70–1050 m/z in DDA mode.

Raw data were preprocessed by Progenesis QI (Waters Corporation, Milford, MA, USA) to generate a data matrix, from which internal standard peaks and false positives were removed. Metabolites were identified against the Human Metabolome Database (HMDB), Metabolite Link (METLIN), and the Majorbio Database (MJDB). The processed matrix was uploaded to the Majorbio Cloud Platform, filtered (features detected in ≥80% of samples per group; missing values filled with the minimum), sum-normalized, and QC-RSD > 30% variables removed. After log10 transformation, multivariate analyses were performed using the R package ‘ropls’ (v1.6.2). Partial least-squares discriminant analysis (PLS-DA) was first applied to visualize the overall metabolic profile separation among the three groups (CON, GAA, and GCSH). Subsequently, orthogonal PLS-DA (OPLS-DA) was performed for pairwise group comparisons to maximize between-group separation and identify differential metabolites. The quality of both PLS-DA and OPLS-DA models was evaluated using R^2^Y (goodness-of-fit) and Q^2^ (predictive ability, derived from 7-fold cross-validation). Model overfitting was assessed by permutation tests (200 permutations). Differential metabolites were selected by Variable Importance in Projection (VIP) > 1 and *p* < 0.05 (*t*-test), and KEGG enrichment was performed using ‘scipy.stats’.

### 2.8. Statistical Analysis

Data were analyzed using the MIXED procedure of SAS (version 9.4, SAS Institute Inc., Cary, NC, USA), with treatment, day (as a categorical fixed effect), and treatment × day interaction as fixed effects. The individual animal served as the experimental unit for all growth performance, nutrient digestibility, serum biochemical, and microbiome/metabolomic analyses. The animal was included as a random intercept to account for individual baseline variation and within-animal correlation across repeated measurements. The covariance structure was selected based on the lowest Akaike information criterion (AIC) among the candidate structures (compound symmetry, AR(1), unstructured, and Toeplitz). Normality of the conditional (studentized) residuals was assessed using normal Q–Q plots and the Shapiro–Wilk test. No outliers were detected based on graphical examination of studentized residuals, and no data points were excluded from the analyses. As the treatment × day interaction was not significant (*p* = 0.92), treatment main-effect means were compared using Tukey’s HSD.

## 3. Results

### 3.1. Growth Performance

The treatment × day interactions for all growth parameters (BW, ADG, DMI, and FCR) were not significant (*p* > 0.05 for all; Table 2). No significant differences were detected between the two supplemented groups (GAA and GCSH) for any growth parameter (*p* > 0.05 for all ADG, DMI, and FCR). However, both supplemented groups had higher ADG and DMI, and lower FCR, than the CON group across all measurement intervals (*p* < 0.05 for all; Table 2).

### 3.2. Apparent Total-Tract Digestibility

Apparent total-tract digestibility of dry matter (DMD), neutral detergent fiber (NDFD), and acid detergent fiber (ADFD) were not affected by treatment (*p* > 0.05 for all; Table 3). In contrast, crude protein digestibility (CPD) differed among groups (*p* = 0.04), with the GCSH group showing the highest CPD, followed by the CON and GAA groups (Table 3).

### 3.3. Serum Biochemical and Antioxidant Parameters

Serum GLU, ALB, GLB, and TC did not differ among groups (*p* > 0.05 for all; Table 4). Serum TP and BUN were affected by treatment (*p* = 0.03 and *p* = 0.01, respectively), with TP higher and BUN lower in both supplemented groups than in CON. For the antioxidant parameters, T-AOC, SOD, and CAT were higher, while MDA was lower, in both supplemented groups compared with CON (*p* < 0.05 for all), with SOD activity being the highest in the GCSH group (Table 4).

### 3.4. Microbial Diversity and Community Assembly

**Figure 1:**
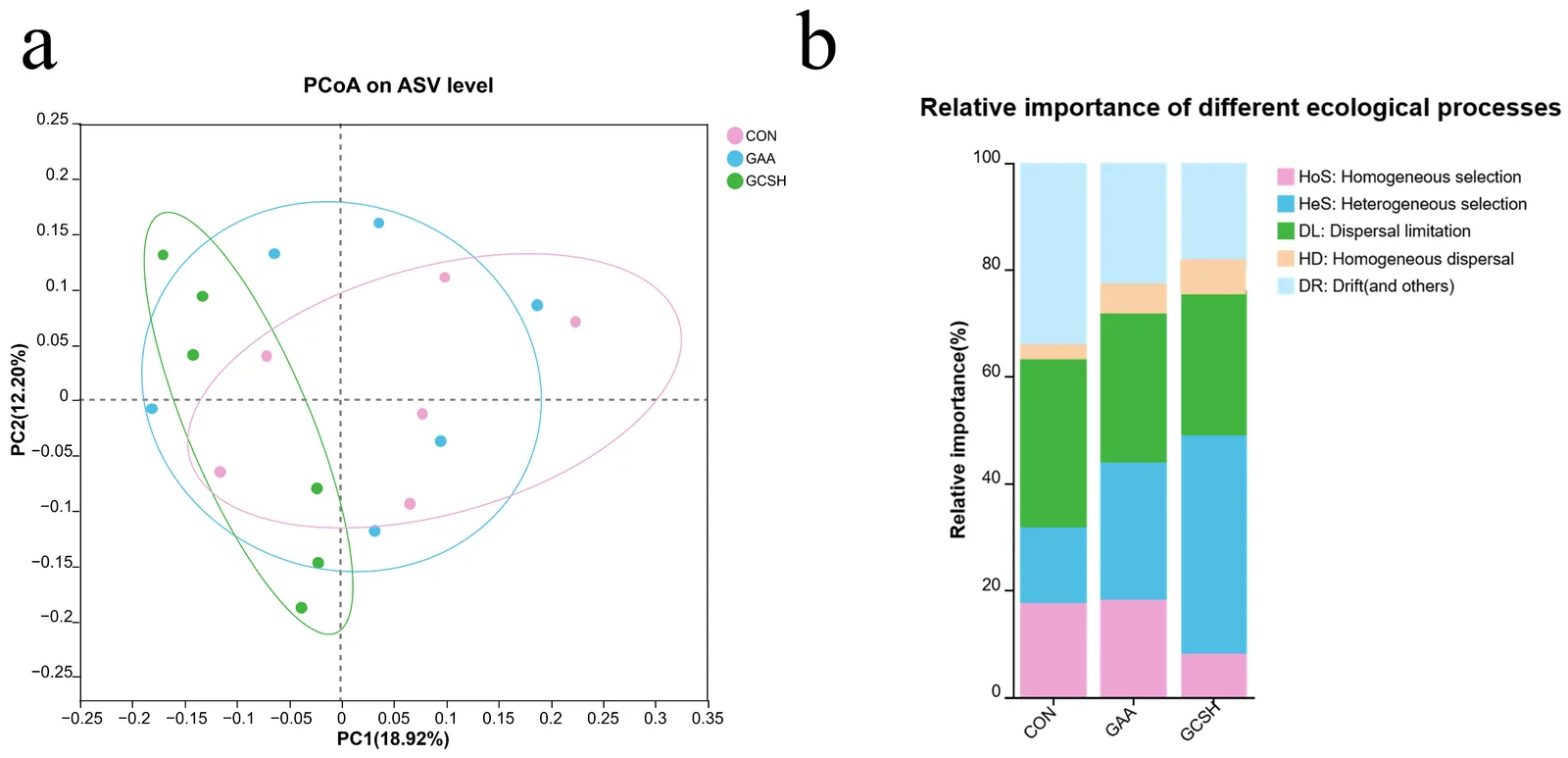
Microbial community structure and ecological assembly mechanisms. (**a**) Principal coordinate analysis (PCoA) based on weighted UniFrac distances at the ASV level. PERMANOVA was used to test the significance of group separation. (**b**) Relative contributions of ecological processes governing microbial community assembly, quantified using the iCAMP framework. HoS, homogeneous selection; HeS, heterogeneous selection; DL, dispersal limitation; HD, homogeneous dispersal; DR, drift and others. CON, control group; GAA, guanidinoacetic acid; GCSH, GAA + cysteamine hydrochloride.

No differences were observed among the CON, GAA, and GCSH groups in any alpha-diversity indices (Chao1, Ace, Shannon, and Simpson; Table 5). However, PCoA based on weighted UniFrac distances suggested a separation among the three groups (Figure 1a). PERMANOVA analysis confirmed a difference in microbial community composition among the three groups (pseudo-F = 2.232, R^2^ = 0.181, *p* = 0.041, 999 permutations). Pairwise comparisons with Benjamini–Hochberg correction showed no differences between any two groups (CON vs. GAA: R^2^ = 0.100, *p* = 0.195; CON vs. GCSH: R^2^ = 0.106, *p* = 0.092; GAA vs. GCSH: R^2^ = 0.120, *p* = 0.032, adjusted *p* = 0.096). The CON group clustered on the right side of PC1, whereas both supplemented groups shifted toward the left side.

To further elucidate the ecological assembly mechanisms underlying these community shifts, the relative contributions of stochastic and deterministic processes were analyzed using the iCAMP framework (Figure 1b). In the CON group, stochastic processes (dispersal limitation, homogeneous dispersal, and drift) collectively dominated assembly (67.7%), with drift as the largest component (34.1%). Deterministic processes accounted for 44.5% in the GAA group, primarily associated with a rise in heterogeneous selection (from 14.1% to 25.9%). The GCSH group exhibited the lowest homogeneous selection (8.3%) and the highest heterogeneous selection (41.1%), with the highest deterministic contribution (49.4%) among all groups.

### 3.5. Taxonomic Composition of Microbiota

**Figure 2:**
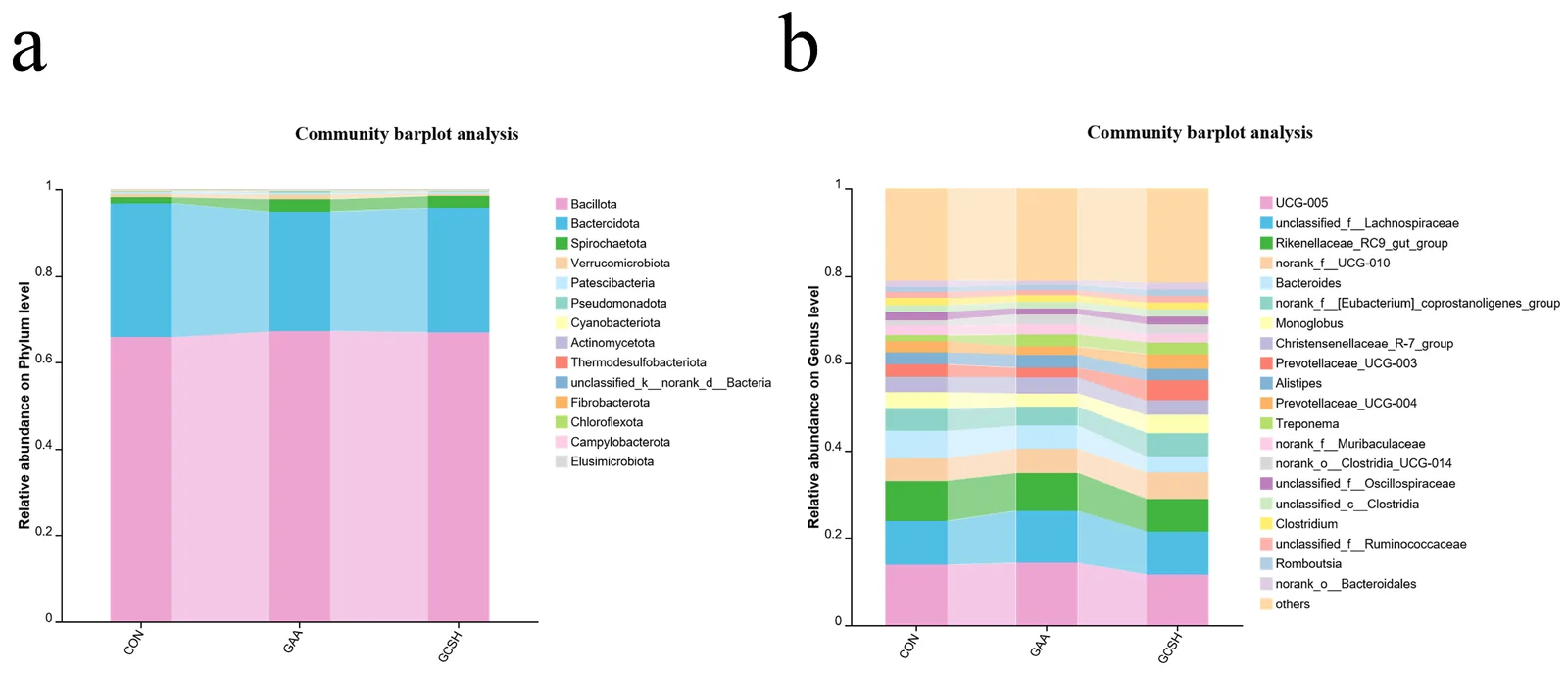
Taxonomic composition of bacterial communities at the phylum and genus levels. (**a**) Phylum-level relative abundances of bacterial communities across the three treatment groups. (**b**) Genus-level relative abundance of the top 20 most abundant genera. Genera with relative abundance < 1% in all groups are grouped as “others”. CON, control group; GAA, guanidinoacetic acid; GCSH, GAA + cysteamine hydrochloride.

At the phylum level, bacterial communities across all groups were dominated by Bacillota and Bacteroidota (Figure 2a). In all groups, Bacillota and Bacteroidota were the absolutely dominant phyla, collectively accounting for over 90% of the total relative abundance across all groups. Collectively, although the dominant phyla remained largely consistent across treatments, GAA and GCSH supplementation shifted the community toward an increased Bacillota-to-Bacteroidota ratio. 

At the genus level, the community was dominated by *UCG-005 (Uncultured Clostridium group 5)*, *unclassified_f_ Lachnospiraceae*, *Rikenellaceae_RC9_gut_group*, and *norank_f_UCG-010 (Uncultured Clostridium group 10)* (Figure 2b). *UCG-005* remained stable between CON and GAA and was lower in GCSH. *Unclassified_f_Lachnospiraceae* increased, whereas *Rikenellaceae_RC9_gut_group* decreased in both supplemented groups. *Bacteroides* declined in GCSH with partial recovery in GAA. *Prevotellaceae_UCG-003* and *Prevotellaceae_UCG-004* showed a distinct pattern, increasing from CON to GCSH, then decreasing sharply in GAA. Several genera, including *norank_f_UCG-010*, *Christensenellaceae_R-7_group*, *Alistipes*, and *Clostridium*, were largely unaffected.

### 3.6. Differential Taxa and Community Dynamics

**Figure 3:**
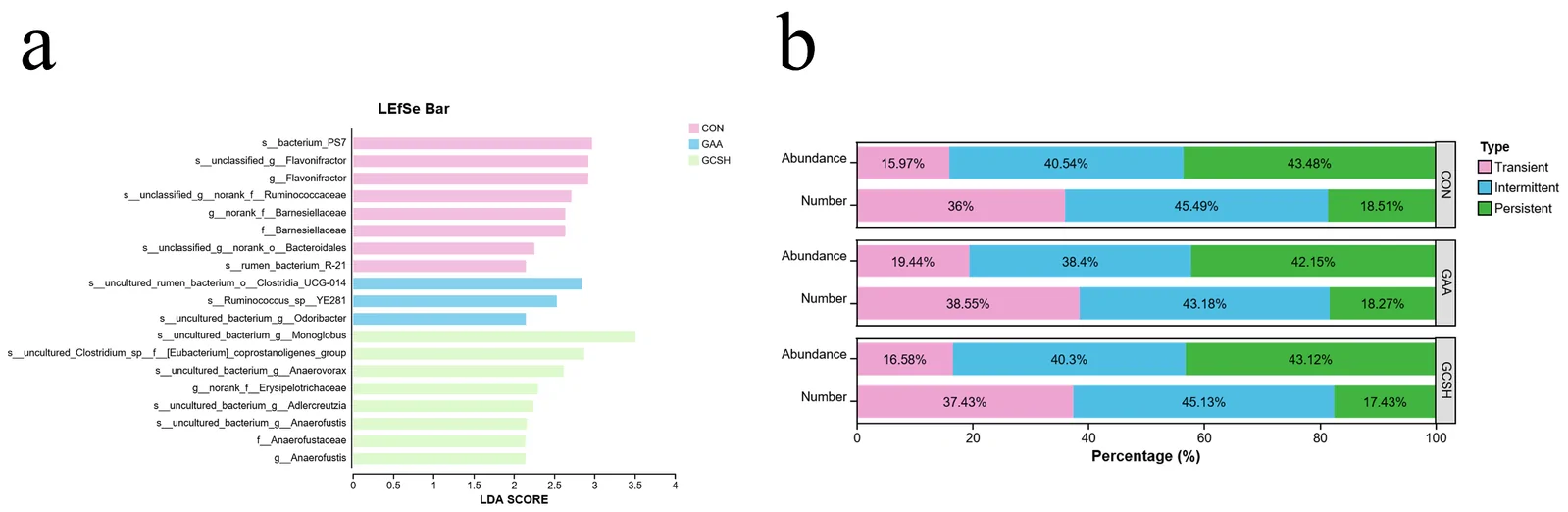
Differential taxa and species persistence patterns of bacterial communities. (**a**) Linear discriminant analysis effect size (LEfSe) identifying differentially abundant taxa among CON, GAA, and GCSH groups. Only taxa with LDA scores of >2 and *p* < 0.05 are shown. (**b**) Classification of all ASVs into transient, intermittent, and persistent categories based on their occurrence frequency across samples within each group. CON, control group; GAA, guanidinoacetic acid; GCSH, GAA + cysteamine hydrochloride; ASV, amplicon sequence variant; LDA, linear discriminant analysis.

LEfSe analysis identified 19 differential taxa across the three groups (LDA score > 2, *p* < 0.05; Figure 3a). The robustness of these LDA scores was evaluated via 999 bootstrap resamplings as implemented in the LEfSe workflow. In the CON group, Flavonifractor (LDA = 2.92) was enriched. The norank_f_Ruminococcaceae (LDA = 2.71) and Barnesiellaceae (LDA = 2.64) were also enriched in the CON group. Taxa enriched in the GAA group comprised only three biomarkers: Ruminococcus_sp._YE281 (LDA = 2.53), Odoribacter (LDA = 2.15), and norank_o_Clostridia_UCG-014 (LDA = 2.84). The GCSH group exhibited enrichment of taxa including Monoglobus (highest LDA = 3.51), Anaerovorax (LDA = 2.62), Anaerofustis (family/genus, LDA = 2.14–2.16), norank_f_Erysipelotrichaceae (LDA = 2.30), and norank_f_[Eubacterium]_coprostanoligenes_group (LDA = 2.87). Beyond taxonomic shifts, the ecological dynamics underlying these community changes were also examined. To further characterize the temporal occupancy of the fecal microbiome, all ASVs were classified into transient, intermittent, and persistent categories based on their occurrence frequency across samples (Figure 3b). The GAA group exhibited a marked increase in the abundance of transient species (19.44%) compared with the CON (15.97%) and GCSH (16.58%) groups, accompanied by a concurrent decrease in the intermittent fraction.

### 3.7. Metabolic Profiling and Chemical Class Distribution

**Figure 4:**
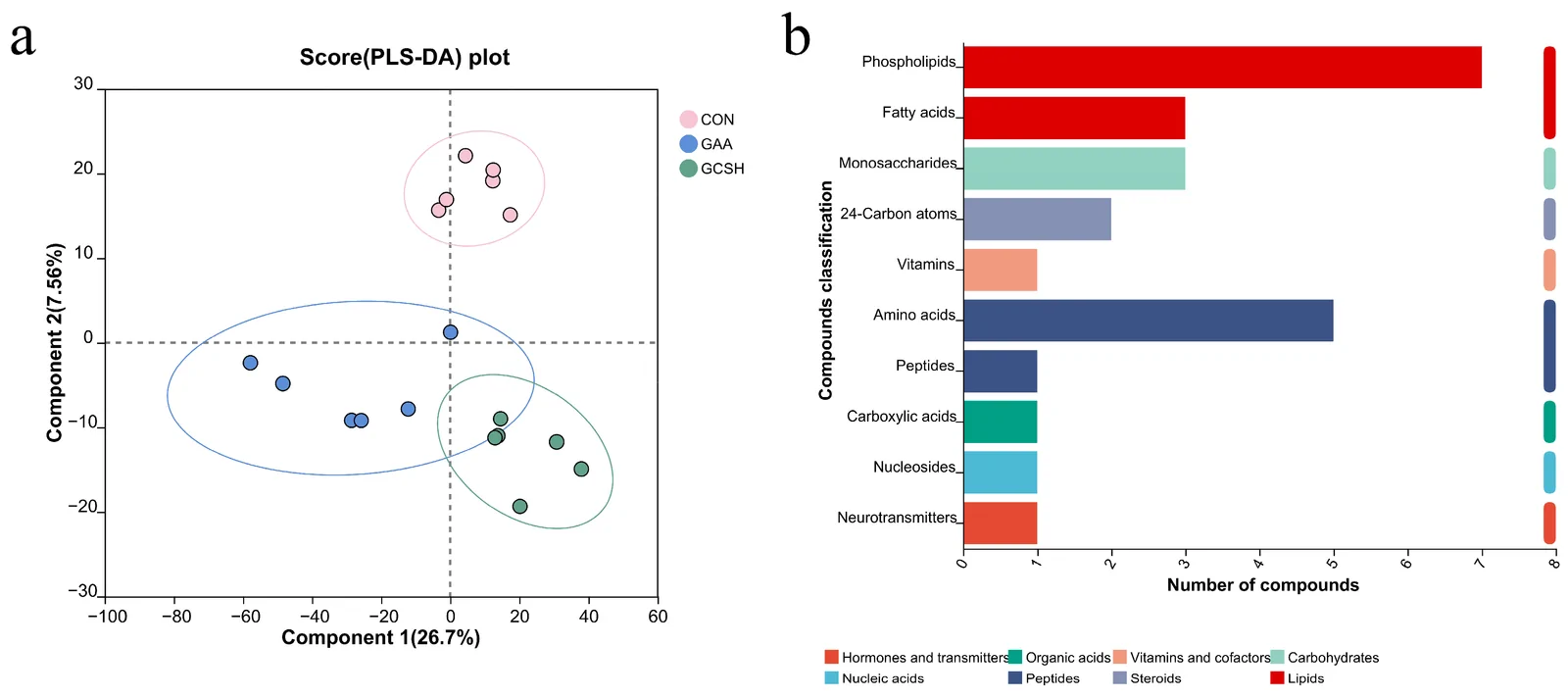
Differential metabolite profiling and chemical class distribution. (**a**) PLS-DA score plot showing the separation of serum metabolomic profiles among CON, GAA, and GCSH groups. (**b**) Classification of differential metabolites (VIP > 1, *p* < 0.05) into chemical classes. CON, control group; GAA, guanidinoacetic acid; GCSH, GAA + cysteamine hydrochloride; PLS-DA, partial least-squares discriminant analysis; VIP, variable importance in projection. The PLS-DA model for the three-group comparison showed good predictive ability (R^2^Y = 0.995, Q^2^ = 0.816), and permutation tests (200 permutations) confirmed the absence of overfitting (*p* < 0.05 for 0/200 permutations).

Serum metabolomics profiles were first assessed by PLS-DA (Figure 4a), which suggested a separation between the CON and treatment groups. PC1 and PC2 explained 26.7% and 7.56% of the total variance, respectively (cumulative: 34.26%). The PLS-DA model showed excellent predictive ability (R^2^Y = 0.995, Q^2^ = 0.816), and permutation tests (200 permutations) confirmed the absence of overfitting (*p* < 0.05 for 0/200 permutations). To characterize the chemical nature of the discriminating metabolites, the 25 differential metabolites (VIP > 1, *p* < 0.05) were classified into 10 chemical classes (Figure 4b). Lipids were the most abundant category, with phospholipids (7 metabolites), fatty acids (3), and 24-carbon atoms (2) collectively accounting for 48% of all differential metabolites. Amino acids (5 metabolites) and peptides (1) represented 24% of the total, while monosaccharides (3), neurotransmitters (1), nucleosides (1), carboxylic acids (1), and vitamins (1) comprised the remaining metabolites.

### 3.8. Serum Metabolomic Profiles and Key Differential Metabolites

**Figure 5:**
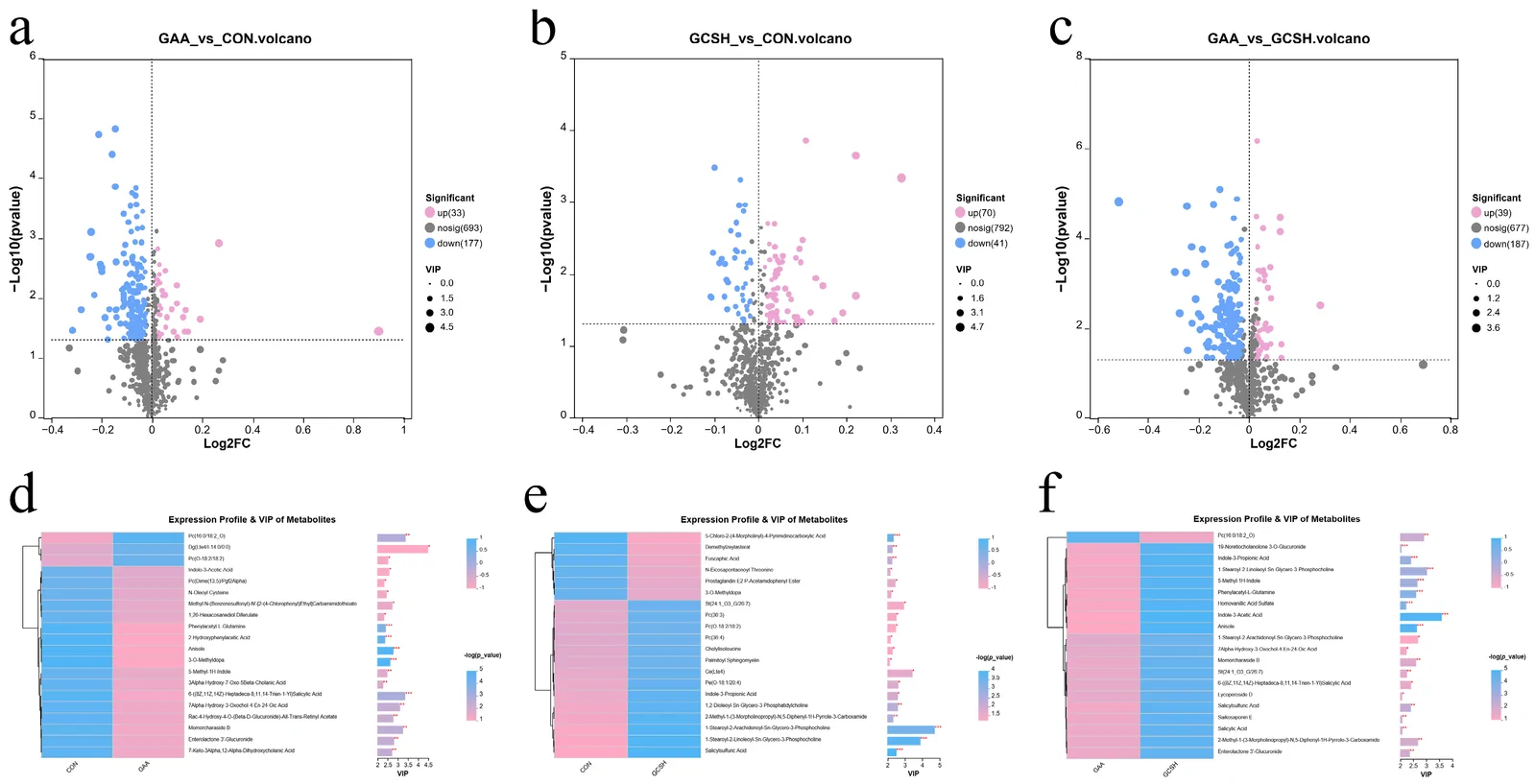
Differential metabolites and VIP analysis. (**a**–**c**) Volcano plots comparing CON vs. GAA, CON vs. GCSH, and GAA vs. GCSH. Red: upregulated; blue: downregulated (log2FC > 1 or <−1, *p* < 0.05); gray: not significant. Dashed lines indicate fold change and significance thresholds. (**d**–**f**) VIP scores of differential metabolites (VIP > 2, *p* < 0.05) from OPLS-DA models comparing the same group pairs. Asterisks denote significance levels: * *p* < 0.05, ** *p* < 0.01, *** *p* < 0.001. CON, control group; GAA, guanidinoacetic acid; GCSH, GAA + cysteamine hydrochloride; FC, fold change; VIP, variable importance in projection; OPLS-DA, orthogonal partial least-squares discriminant analysis.

The OPLS-DA models for pairwise comparisons also demonstrated excellent predictive ability. For the CON vs. GAA model, R^2^X = 0.507, R^2^Y = 0.998, and Q^2^ = 0.649; for the CON vs. GCSH model, R^2^X = 0.292, R^2^Y = 0.995, and Q^2^ = 0.601; and for the GAA vs. GCSH model, R^2^X = 0.514, R^2^Y = 0.999, and Q^2^ = 0.706. Permutation tests (200 permutations) confirmed the absence of overfitting for all three models (*p* < 0.05 for 0/200 permutations). In serum metabolomics, volcano plot analysis revealed that, compared with the CON group, the GAA group exhibited 210 differentially abundant metabolites, of which 33 were upregulated and 177 downregulated (Figure 5a). The number of differential metabolites was lower in the GCSH vs. CON comparison (Figure 5b), with 111 identified (70 upregulated, 41 downregulated). When comparing the GCSH group with the GAA group, 226 differentially abundant metabolites were identified, comprising 39 upregulated and 187 downregulated metabolites (Figure 5c).

Focusing on the GAA vs. CON comparison, VIP analysis (VIP > 2, *p* < 0.05) identified 20 differential metabolites (Figure 5d), of which 3 lipid-related species were upregulated, and 17 were downregulated. Key downregulated metabolites included phenylalanine-derived phenylacetyl-L-glutamine (VIP = 2.40, *p* < 0.05), 2-hydroxyphenylacetic acid (VIP = 2.36, *p* < 0.05), tryptophan-derived indole-3-acetic acid (VIP = 2.57, *p* = 0.035), and 5-methyl-1H-indole (VIP = 2.47, *p* = 0.009). In marked contrast, the CON vs. GCSH comparison (Figure 5e) showed a strikingly different pattern: among the 20 differential metabolites (VIP > 2, *p* < 0.05), 14 were upregulated, and only 6 were downregulated. Key upregulated metabolites included phospholipids (e.g., 1-stearoyl-2-arachidonoyl-sn-glycero-3-phosphocholine, VIP = 4.71; 1-stearoyl-2-linoleoyl -sn-glycero-3-phosphocholine, VIP = 3.88), indole-3-propionic acid (IPA; VIP = 2.57, *p* = 0.012), a microbial tryptophan metabolite reported to possess antioxidant properties in non-ruminant models, and salicylsulfuric acid (VIP = 2.50, *p* = 0.00014), which has been associated with anti-inflammatory activity in other contexts, while a prostaglandin E2 derivative (VIP = 2.45, *p* = 0.021) was downregulated. Direct comparison between the two supplemented groups (GAA vs. GCSH, Figure 5f) further refined this distinction: among 20 differential metabolites (VIP > 2, *p* < 0.05), 19 were upregulated and only 1 [Pc(16:0/18:2_O), VIP = 2.91] was downregulated in GCSH relative to GAA. Key upregulated metabolites included tryptophan-derived indole-3-acetic acid (VIP = 3.59, *p* < 0.001), phenylalanine-derived phenylacetyl-L-glutamine (VIP = 2.61, *p* < 0.001), and phospholipids such as 1-stearoyl-2-linoleoyl PC (VIP = 3.02) and 1-stearoyl-2-arachidonoyl PC (VIP = 2.67).

### 3.9. KEGG Pathway Enrichment and Topological Analysis

**Figure 6:**
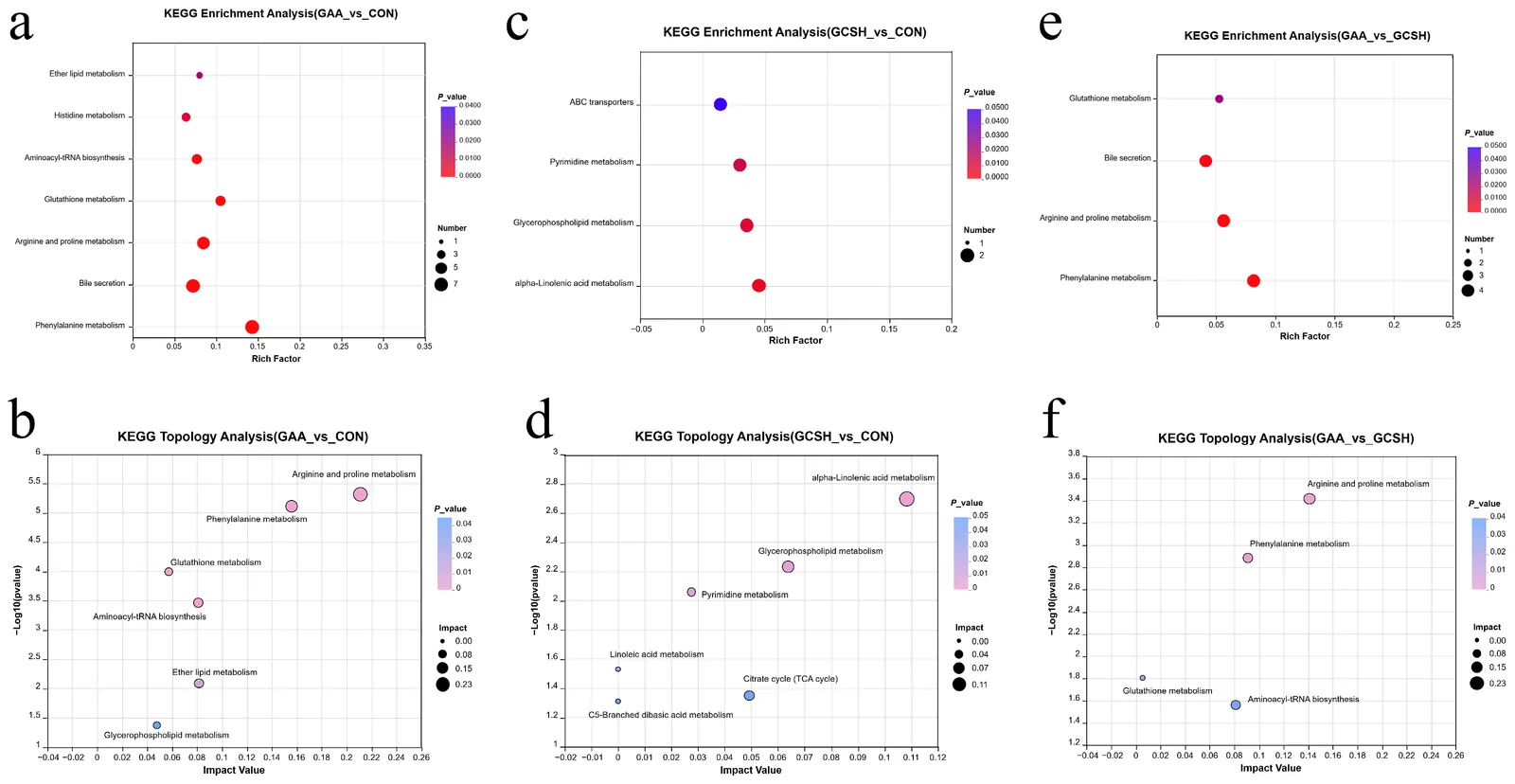
KEGG pathway enrichment and topological analysis of differential metabolites among CON, GAA, and GCSH groups. (**a**,**c**,**e**) Enrichment analysis showing enriched pathways (*p* < 0.05) with the rich factor on the x-axis and pathway categories on the y-axis for GAA vs. CON (**a**), GCSH vs. CON (**c**), and GAA vs. GCSH (**e**). (**b**,**d**,**f**) Topological analysis (impact value) of key pathways identified in the three-group comparison for GAA vs. CON (**b**), GCSH vs. CON (**d**), and GAA vs. GCSH (**f**). Larger impact values indicate greater pathway centrality and regulatory importance. Circle size represents the number of enriched metabolites in each pathway. Color gradient represents *p*-values (red: lower *p*-value; blue: higher *p*-value). KEGG, Kyoto Encyclopedia of Genes and Genomes; CON, control group; GAA, guanidinoacetic acid; GCSH, GAA + cysteamine hydrochloride.

To translate these metabolite-level changes into functional pathways, KEGG enrichment analysis was performed on the differential metabolites between the CON group and the GAA group, identifying seven enriched pathways (*p* < 0.05; Figure 6a). Phenylalanine metabolism, arginine and proline metabolism, aminoacyl-tRNA biosynthesis, glutathione metabolism, and histidine metabolism were among the enriched pathways. Ether lipid metabolism and bile secretion were also among the enriched pathways. Complementing the enrichment analysis, topological analysis of these pathways (Figure 6b) indicated phenylalanine metabolism as the primary regulatory hub, with ether lipid metabolism and aminoacyl-tRNA biosynthesis serving as secondary hubs. Glutathione metabolism and glycerophospholipid metabolism showed a moderate impact trend.

In the CON vs. GCSH pairwise contrast, four KEGG pathways were enriched (Figure 6c): glycerophospholipid metabolism, α-linolenic acid metabolism, pyrimidine metabolism, and bile secretion. Topological analysis (Figure 6d) identified α-linolenic acid metabolism as the primary metabolic hub, with glycerophospholipid metabolism showing moderate impact. The TCA cycle, linoleic acid metabolism, and C5-branched dibasic acid metabolism also showed impact.

Direct comparison between GAA and GCSH (Figure 6e) revealed four enriched pathways: phenylalanine metabolism, arginine and proline metabolism, glutathione metabolism, and bile secretion. Topological analysis (Figure 6f) indicated arginine and proline metabolism as the primary differentiating hub for this comparison. Glutathione metabolism and aminoacyl-tRNA biosynthesis also showed impact. 

## 4. Discussion

The present study provides a multi-omics characterization of the complementary effects of GAA and CSH co-supplementation in Simmental beef cattle. Both GAA alone and GAA + CSH improved growth performance and feed efficiency. GAA supplementation was primarily associated with enhanced nitrogen retention and lower levels of phenylalanine- and tryptophan-derived metabolites, whereas CSH co-supplementation conferred additional layers of modulation: enhanced antioxidant enzyme activities; elevated circulating levels of indole-3-propionic acid and phospholipids; enrichment of specific taxa with documented or predicted metabolic functions, including *Adlercreutzia*—a genus capable of metabolizing isoflavones to equol, a metabolite with antioxidant activity [18,19]—*Anaerofustis* and *Anaerovorax*—potential producers of acetate and butyrate [20,21]—and *Monoglobus*—a pectin-fermenting genus associated with fiber utilization [22]. Integrated with iCAMP analysis, these findings suggest that GAA and CSH operate through complementary mechanisms. GAA primarily drives nitrogen metabolism and growth, while CSH reinforces antioxidant defense and reshapes fecal microbial assembly by relieving homogenizing selection pressure. This integrated perspective moves beyond describing individual treatment effects toward a mechanistic understanding of how these two additives jointly influence host metabolism and intestinal ecology.

### 4.1. Growth Performance and Nitrogen Utilization

The growth response to GAA is consistent with previous reports [23,24] and was particularly pronounced in lighter animals (285 kg) during rapid growth, when creatine precursor demand was high. Metabolomic evidence was consistent with the interpretation that GAA was associated with arginine being directed toward creatine synthesis, as suggested by enriched arginine–proline metabolism and lower blood urea nitrogen levels, consistent with improved nitrogen retention [25]. Enhanced aminoacyl-tRNA biosynthesis was further consistent with protein synthesis, consistent with elevated serum total protein. The increased Bacillota-to-Bacteroidota ratio is also consistent with the observed improved energy harvest and feed efficiency [26]. Notably, GCSH showed superior crude protein digestibility over GAA alone, suggesting that CSH further enhanced protein utilization [27]. The enhanced crude protein digestibility in the GCSH group, the only digestibility parameter affected, is consistent with reports that CSH supplementation increases CP digestibility in cattle and improves nitrogen retention in ruminants [28]. This effect may involve somatostatin inhibition leading to enhanced digestive enzyme secretion, as well as reduced fecal and urinary nitrogen losses. The enrichment of *Anaerovorax* in the GCSH group may also be relevant, as this genus is associated with amino acid metabolism in the rumen. These interpretations remain correlational and require direct validation in cattle, but collectively they suggest that CSH co-supplementation may enhance protein utilization through improved digestive efficiency and reduced nitrogen loss.

### 4.2. Antioxidant and Immunomodulatory Benefits

The GCSH group exhibited the highest T-AOC, SOD, and CAT activities and reduced the level of MDA, with glutathione metabolism enrichment suggesting enhanced glutathione synthetase [29]. Mechanistically, CSH is thought to activate Nrf2/ARE, potentially upregulating antioxidant enzymes, and may suppress NF-κB [6,7], consistent with lower levels of phenylalanine-derived inflammatory metabolites in the GCSH group. CSH has been reported to activate the GH–IGF-1 axis via somatostatin depletion [5], which may explain the numerically higher early-phase ADG. Although serum homocysteine was not measured, the GCSH group performed at least as well as GAA across all antioxidant indicators, suggesting that CSH may provide complementary protection, although this interpretation is primarily based on non-ruminant models and requires direct validation in cattle.

### 4.3. Fecal Microbiome and Serum Metabolome Remodeling

Despite unchanged alpha diversity, the PCoA revealed moderate restructuring of the fecal microbial community in both supplemented groups. The GCSH group exhibited a higher relative abundance of several genera with documented or predicted metabolic functions. *Adlercreutzia,* a genus reported to metabolize dietary polyphenols in microbial culture studies [18], was specifically enriched in GCSH. *Monoglobus*, which has been linked to pectin degradation and fiber utilization, also showed higher abundance in this group [22]. In addition, *Anaerofustis* and *Anaerovorax*, both considered potential butyrate producers, were enriched in GCSH [20,21]. In contrast, the CON group was characterized by the enrichment of *Flavonifractor*, a genus that can indirectly alleviate inflammation in ruminant animals [30].

The ecological assembly mechanisms underlying these compositional shifts were further elucidated using the iCAMP framework. The iCAMP analysis revealed distinct ecological assembly mechanisms among the three groups. Stochastic processes dominated the CON group, characteristic of weak environmental filtering and suggesting that community composition was largely driven by random dispersal and neutral dynamics rather than strong host selection [31]. GAA supplementation was associated with a shift toward deterministic processes, primarily through increased heterogeneous selection. The GCSH group exhibited the lowest homogeneous selection and the highest heterogeneous selection among all groups, resulting in the highest deterministic contribution, yet achieved through a fundamentally different balance. Homogeneous selection tends to reduce community variation by favoring the same taxa across replicate individuals, whereas heterogeneous selection promotes divergence by favoring different taxa in different individuals [32]. The marked reduction in homogeneous selection in GCSH, coupled with enhanced heterogeneous selection, suggests that CSH co-supplementation relaxed homogenizing pressure, potentially allowing for greater niche diversification. High heterogeneous selection with markedly reduced homogenizing constraint, consistent with the hypothesis that CSH supplementation may relieve homogenizing selection pressure, potentially allowing for the establishment of beneficial taxa through niche diversification [32]. However, this interpretation remains correlational and requires direct experimental validation. Supporting this view, species persistence analysis showed that GAA was associated with an increased proportion of transient species compared with CON and GCSH, suggesting that GAA-associated deterministic selection may have promoted taxa that failed to establish persistent populations, potentially contributing to ecological disturbance. In contrast, GCSH restored transient proportions to levels similar to CON, suggesting a more stabilized core microbiota that may be functionally relevant for consistent host–microbe interactions [33].

These microbial shifts were accompanied by corresponding serum metabolomic changes. In the GAA vs. CON comparison, phenylalanine- and tryptophan-derived metabolites, including phenylacetyl-L-glutamine and indole-3-acetic acid, were present at lower levels. In contrast, the GCSH vs. CON comparison showed that 14 of 20 differential metabolites were present at higher levels, most notably indole-3-propionic acid (IPA), a microbial tryptophan metabolite reported to activate the pregnane X receptor and maintain intestinal barrier integrity in mouse models [34]. The concurrent enrichment of *Adlercreutzia* and IPA elevation in the GCSH group offers a correlational pattern that may hypothetically link the fecal microbiota to host antioxidant status; however, this interpretation requires direct validation in bovine models. Nevertheless, the observed IPA elevation in the GCSH group warrants further investigation into its potential biological significance in beef cattle.

At the pathway level, phenylalanine metabolism consistently emerged as the most robust pathway differentiating the three groups across all comparisons. The consistent downregulation of phenylacetyl-L-glutamine and 2-hydroxyphenylacetic acid in both supplemented groups—particularly in GCSH—is consistent with reduced inflammatory burden, as these metabolites are established markers of inflammatory stress [35]. Phospholipids constituted the largest class of differential metabolites, and both glycerophospholipid and ether lipid metabolism were enriched. This phospholipid remodeling may reinforce membrane integrity and optimize nutrient transport, aligning with the improved growth performance observed [36]. Together, these pathway-level changes suggest that GAA is primarily associated with nitrogen and energy metabolism, while CSH co-supplementation is associated with reinforced antioxidant defense systems and a more stable microbial community structure with potential functional benefits.

### 4.4. Integrated Mechanistic Model and Implications

**Figure 7:**
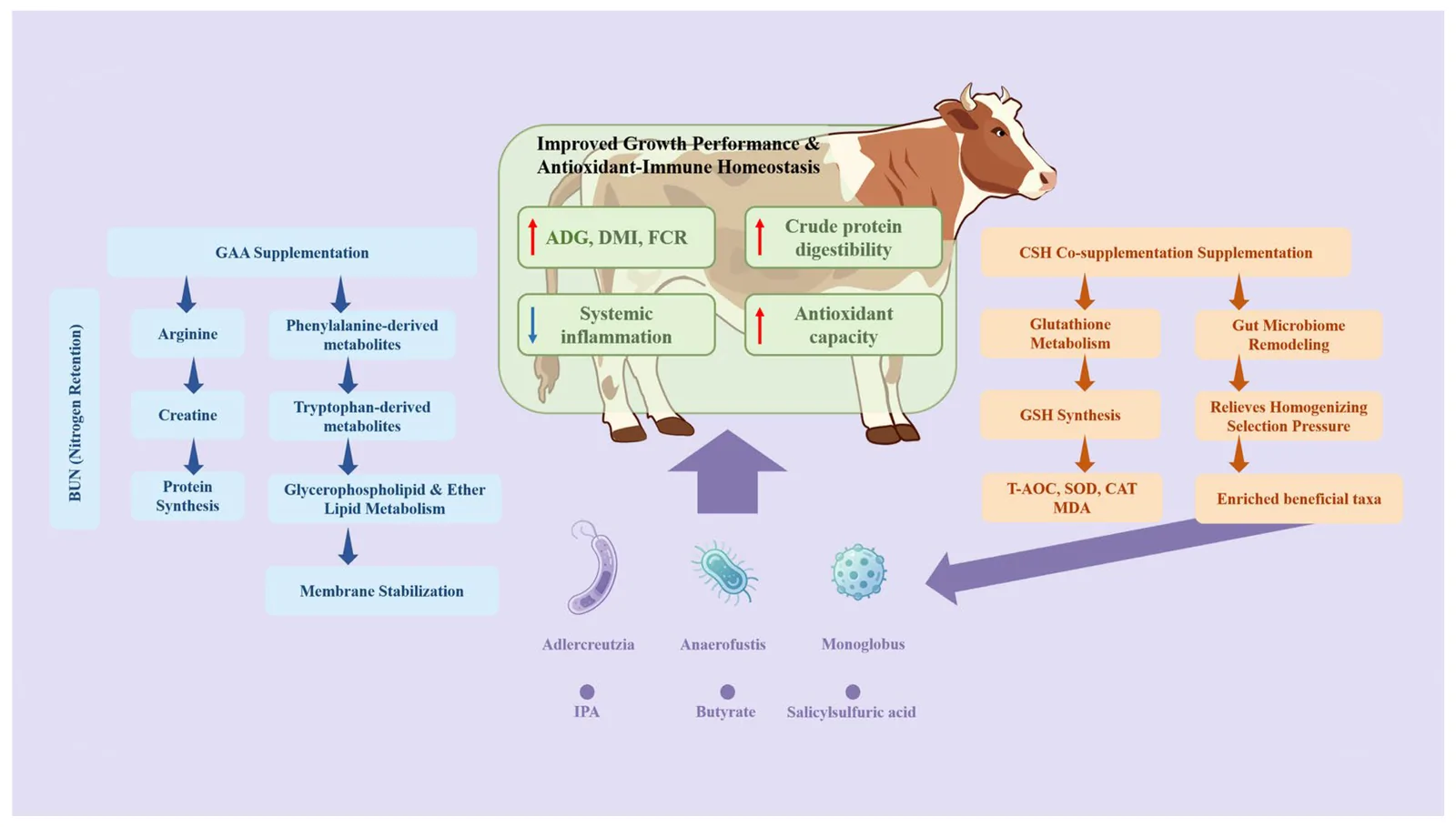
Proposed mechanistic model for the complementary effects of GAA and CSH co-supplementation. GAA is hypothesized to enhance nitrogen utilization (arginine → creatine), promote protein synthesis, suppress inflammatory metabolites, and initiate membrane phospholipid remodeling. CSH may add complementary layers: glutathione-mediated antioxidant defense (T-AOC, SOD, CAT ↑; MDA ↓), fecal microbiome remodeling (relieving homogenizing selection pressure), and enrichment of beneficial taxa (*Adlercreutzia*, *Anaerofustis*, and *Monoglobus*) potentially producing functional metabolites (IPA, butyrate, salicylsulfuric acid). These pathways are hypothesized to converge in contributing to improved growth performance (ADG, DMI, FCR), crude protein digestibility, and systemic antioxidant–immune homeostasis, based on correlational multi-omics observations. GAA, guanidinoacetic acid; CSH, cysteamine hydrochloride; ADG, average daily gain; DMI, dry matter intake; FCR, feed conversion ratio; T-AOC, total antioxidant capacity; SOD, superoxide dismutase; CAT, catalase; MDA, malondialdehyde; IPA, indole-3-propionic acid. Arrows indicate hypothesized or potentially contributory relationships based on correlational observations.

Based on the correlational multi-omics data, a working model is proposed (Figure 7) wherein GAA is hypothesized to act through three pathways: (1) upregulation of creatine precursor synthesis, diverting arginine toward creatine and potentially improving nitrogen retention; (2) downregulation of phenylalanine- and tryptophan-derived metabolites; and (3) upregulation of membrane phospholipid remodeling. CSH may add two additional layers: (4) enhanced glutathione-mediated antioxidant defense; (5) reshaping of fecal microbial assembly by relieving homogenizing selection pressure. These potentially create a permissive environment for beneficial taxa (*Adlercreutzia*, *Anaerofustis*, and *Monoglobus*) that may produce antioxidant and anti-inflammatory metabolites (IPA, salicylsulfuric acid, butyrate). The multi-omics integration elucidates the microbe–metabolite–antioxidant axis, providing a foundation for future mechanistic studies. 

Several methodological constraints should be acknowledged. The most notable limitation is the absence of a CSH-only treatment group in our experimental design. With only the CON, GAA, and GCSH groups, the two-supplemented-group comparison effectively demonstrates additional effects conferred by CSH co-supplementation on top of GAA. However, this design does not allow us to unambiguously partition the observed GCSH effects into CSH-specific actions, additive GAA + CSH effects, or true GAA–CSH interactions. Consequently, the mechanistic inferences, particularly those concerning the specific roles of CSH in fecal microbiome assembly (e.g., enrichment of *Adlercreutzia*, *Anaerofustis*, and *Monoglobus*) and the elevation of indole-3-propionic acid, should be interpreted as associative and hypothesis-generating rather than as definitive causal attributions to CSH per se. Additionally, samples were collected only at the endpoint (day 60), and no samples were collected at day 0 or day 30. A time-course sampling design would have allowed us to monitor the dynamic trajectory of microbiome and metabolome remodeling in response to GAA and CSH supplementation and to distinguish transient fluctuations from stable treatment effects. Although our endpoint data revealed significant differences among treatments, the absence of longitudinal sampling limits our ability to interpret the temporal dynamics of these changes or to directly attribute them to the selection pressure exerted by the additives. Future studies incorporating multiple time-point sampling (e.g., day 0, day 30, and day 60), fully factorial 2 × 2 designs (CON, GAA, CSH, and GAA + CSH) with adequate statistical power are warranted to validate the observed shifts, confirm the robustness of these findings, and rigorously dissect the individual and interactive contributions of these two additives.

## 5. Conclusions

In conclusion, dietary supplementation with GAA alone or in combination with CSH for 60 days improved growth performance and feed efficiency in Simmental beef cattle. While both GAA and GCSH were associated with improved nitrogen utilization and phospholipid remodeling, CSH co-supplementation was uniquely associated with enhanced systemic antioxidant capacity and an altered fecal microbial ecological assembly pattern by relieving homogenizing selection pressure, a pattern not observed with GAA alone. Although GCSH numerically improved growth metrics compared with GAA alone, these differences did not reach statistical significance. Its distinct capacity to enhance systemic antioxidant capacity and reshape fecal ecological assembly, a distinct profile not conferred by GAA alone, supports the application of GCSH as a complementary feed additive strategy for improving both productivity and health status in beef cattle.

## Figures and Tables

**Table 1:** Ingredients and chemical composition of the basal diet (dry-matter basis).

Composition	
Ingredients (%)	
Whole-plant corn silage	30.0
Dry corn straw	15.0
Ground corn	30.0
Soybean meal (43% CP)	10.5
DDGS	8.0
Wheat bran	3.5
Limestone	1.2
Dicalcium phosphate	0.5
NaCl	0.5
Vitamin-mineral premix ^1^	0.5
Sodium bicarbonate	0.3
Total	100.00
Nutrition level	
Dry matter, %	55.98
ME, MJ/kg DM ^2^	12.08
Crude protein, %	13.21
Neutral detergent fiber, %	35.1
Acid detergent fiber, %	22.1
Ca, %	0.55
P, %	0.35

^1^ Provided per kg of diet dry matter (DM): vitamin A = 8000 IU, vitamin D_3_ = 2000 IU, vitamin E = 50 IU, Cu = 8 mg, Fe = 50 mg, Mn = 30 mg, Zn = 40 mg, Se = 0.15 mg, I = 0.5 mg, Co = 0.1 mg. ^2^ DM = dry matter; ME = metabolizable energy; CP = crude protein; NDF = neutral detergent fiber; ADF = acid detergent fiber; DDGS = distillers dried grains with solubles. ME was a calculated value; all other chemical composition values were determined by laboratory analysis.

**Table 2:** Effects of dietary GAA and GAA combined with CSH on growth performance, feed intake, and feed efficiency in Simmental beef cattle.

	Treatment				*p*-Value		
Item	CON	GAA	GCSH	SEM	Treatment	Time	Interaction
BW, kg							
0 d	286.23	283.20	284.60	5.232	0.84	<0.0001	0.92
30 d	315.93 ^b^	322.20 ^a^	323.06 ^a^	3.645	0.02		
60 d	342.93 ^b^	358.56 ^a^	360.83 ^a^	3.886	0.01		
ADG, kg/d							
0–30 d	0.99 ^b^	1.30 ^a^	1.28 ^a^	0.034	<0.0001	0.12	0.59
31–60 d	0.90 ^b^	1.21 ^a^	1.26 ^a^	0.078	0.01		
0–60 d	0.95 ^b^	1.26 ^a^	1.27 ^a^	0.035	<0.0001		
DMI, kg							
0–30 d	9.26 ^b^	11.00 ^a^	10.70 ^a^	1.028	0.03	0.51	0.26
31–60 d	8.87 ^b^	10.80 ^a^	10.30 ^a^	1.007	0.03		
0–60 d	9.06 ^b^	10.09 ^a^	10.00 ^a^	0.976	0.04		
FCR							
0–30 d	9.35 ^a^	8.46 ^b^	8.36 ^b^	1.857	0.01	0.23	0.58
31–60 d	9.86 ^a^	8.92 ^b^	8.17 ^b^	2.652	0.03		
0–60 d	9.54 ^a^	8.01 ^b^	7.87 ^b^	1.462	0.01		

Values are presented as least-squares means with pooled standard error of the mean (SEM). Within each row, means with different superscript letters (a, b) differ significantly (*p* < 0.05); the absence of superscript letters indicates no significant difference (*p* > 0.05). BW = body weight; ADG = average daily gain; DMI = dry matter intake; FCR = feed conversion ratio (feed:gain); CON = control group; GAA = guanidinoacetic acid group; GCSH = GAA + cysteamine hydrochloride group.

**Table 3:** Effects of dietary GAA and GAA combined with CSH on apparent total-tract nutrient digestibility in Simmental beef cattle.

	Treatment				
Item	CON	GAA	GCSH	SEM	*p*-Value
NDFD, %	37.04	39.91	37.21	0.361	0.44
ADFD, %	56.38	57.33	55.77	0.267	0.35
DMD, %	61.35	61.88	63.42	0.153	0.12
CPD, %	59.64 ^b^	58.86 ^b^	63.57 ^a^	0.457	0.04

Values are presented as least-squares means with pooled standard error of the mean (SEM). Within each row, means with different superscript letters (a, b) differ significantly (*p* < 0.05); the absence of superscript letters indicates no significant difference (*p* > 0.05). NDFD = neutral detergent fiber digestibility; ADFD = acid detergent fiber digestibility; DMD = dry matter digestibility; CPD = crude protein digestibility; CON = control group; GAA = guanidinoacetic acid group; GCSH = GAA + cysteamine hydrochloride group.

**Table 4:** Effects of dietary GAA and GAA combined with CSH on serum biochemical parameters and antioxidant status in Simmental beef cattle.

	Treatment				
Item	CON	GAA	GCSH	SEM	*p*-Value
GLU, mmol/L	4.68	4.95	4.70	0.122	0.25
TP, g/L	61.46 ^b^	64.21 ^a^	65.62 ^a^	1.520	0.03
ALB, g/L	34.84	34.23	35.21	1.005	0.78
GLB, g/L	28.62	29.97	30.41	0.985	0.34
BUN, mg/dL	7.52 ^a^	6.67 ^b^	6.30 ^b^	0.479	0.01
TC, mmol/L	2.46	2.54	2.74	0.170	0.50
T-AOC, U/mL	8.280 ^b^	9.79 ^a^	9.99 ^a^	0.186	0.02
MDA, nmol/L	7.39 ^a^	5.05 ^b^	5.35 ^b^	0.363	0.04
SOD, U/mL	161.19 ^b^	182.25 ^a^	188.97 ^a^	7.623	0.01
CAT, U/mL	47.02 ^b^	52.17 ^a^	53.20 ^a^	2.186	0.02

Values are presented as least-squares means with pooled standard error of the mean (SEM). Within each row, means with different superscript letters (a, b) differ significantly (*p* < 0.05); the absence of superscript letters indicates no significant difference (*p* > 0.05). GLU = glucose; TP = total protein; ALB = albumin; GLB = globulin (calculated as TP minus ALB); BUN = blood urea nitrogen; TC = total cholesterol; T-AOC = total antioxidant capacity; MDA = malondialdehyde; SOD = superoxide dismutase; CAT = catalase; CON = control group; GAA = guanidinoacetic acid group; GCSH = GAA + cysteamine hydrochloride group.

**Table 5:** Effects of dietary GAA and GAA combined with CSH on alpha-diversity indices of bacterial communities in Simmental beef cattle.

Item	Treatment			SEM	*p*-Value
	CON	GAA	GCSH		
Chao1	981.36	1053.93	961.73	92.178	0.52
Ace	981.12	1053.51	961.79	92.079	0.52
Shannon	6.25	6.30	6.23	0.195	0.89
Simpson	0.0036	0.0038	0.0038	0.00128	0.92

Values are presented as least-squares means with pooled standard error of the mean (SEM). No superscript letters are present within rows, as no significant differences were detected among treatments for any alpha-diversity index (*p* > 0.05 for all). Alpha-diversity indices were calculated based on amplicon sequence variant (ASV) tables rarefied to 20,000 reads per sample, as described in Section 2.6. CON = control group; GAA = guanidinoacetic acid group; GCSH = GAA + cysteamine hydrochloride group; SEM = standard error of the mean.

## Data Availability

The 16S rRNA sequencing data presented in this study are openly available in the NCBI Sequence Read Archive (SRA) under BioProject accession number PRJNA1507570. You can learn about the original data at https://www.ncbi.nlm.nih.gov/bioproject/PRJNA1507570 (accessed on 13 September 2026). The serum metabolomics data have been deposited in the OMIX database of the National Genomics Data Center (NGDC) under accession number OMIX019384. You can learn about the original data at https://ngdc.cncb.ac.cn/omix/release/OMIX019384 (accessed on 13 September 2026). Both datasets are publicly accessible.

## Abbreviations

The following abbreviations are used in this manuscript:

ADG	average daily gain
AIA	acid-insoluble ash
ASV	amplicon sequence variant
BUN	blood urea nitrogen
CAT	catalase
CON	control group
CP	crude protein
CPD	crude protein digestibility
CSH	cysteamine hydrochloride
DDGS	distillers dried grains with solubles
DL	dispersal limitation
DM	dry matter
DMD	dry matter digestibility
DMI	dry matter intake
DR	drift and others
FCR	feed conversion ratio
GAA	guanidinoacetic acid
GCSH	GAA + cysteamine hydrochloride group
HD	homogeneous dispersal
HeS	heterogeneous selection
HoS	homogeneous selection
IPA	indole-3-propionic acid
KEGG	Kyoto Encyclopedia of Genes and Genomes
LDA	linear discriminant analysis
LEfSe	linear discriminant analysis effect size
MDA	malondialdehyde
ME	metabolizable energy
NDF	neutral detergent fiber
NDFD	neutral detergent fiber digestibility
OPLS-DA	orthogonal partial least-squares discriminant analysis
PCoA	principal coordinate analysis
PERMANOVA	permutational multivariate analysis of variance
PLS-DA	partial least-squares discriminant analysis
QC	quality control
ROS	reactive oxygen species
SEM	standard error of the mean
SOD	superoxide dismutase
T-AOC	total antioxidant capacity
TC	total cholesterol
TMR	total mixed ration
TP	total protein
VIP	variable importance in projection
